# Failure modes and effects analysis (FMEA) for Gamma Knife radiosurgery

**DOI:** 10.1002/acm2.12205

**Published:** 2017-10-29

**Authors:** Andy Yuanguang Xu, Jagdish Bhatnagar, Greg Bednarz, John Flickinger, Yoshio Arai, Jonet Vacsulka, Wenzheng Feng, Edward Monaco, Ajay Niranjan, L. Dade Lunsford, M. Saiful Huq

**Affiliations:** ^1^ Department of Radiation Oncology University of Pittsburgh Cancer Institute Pittsburgh PA USA; ^2^ Department of Neurological Surgery University of Pittsburgh Medical Center Pittsburgh PA USA; ^3^ Department of Radiation Oncology New York Presbyterian Hospital/Columbia University Medical Center New York NY USA

**Keywords:** FMEA, Gamma Knife, quality assurance, radiosurgery

## Abstract

**Purpose:**

Gamma Knife radiosurgery is a highly precise and accurate treatment technique for treating brain diseases with low risk of serious error that nevertheless could potentially be reduced. We applied the AAPM Task Group 100 recommended failure modes and effects analysis (FMEA) tool to develop a risk‐based quality management program for Gamma Knife radiosurgery.

**Methods:**

A team consisting of medical physicists, radiation oncologists, neurosurgeons, radiation safety officers, nurses, operating room technologists, and schedulers at our institution and an external physicist expert on Gamma Knife was formed for the FMEA study. A process tree and a failure mode table were created for the Gamma Knife radiosurgery procedures using the Leksell Gamma Knife Perfexion and 4C units. Three scores for the probability of occurrence (O), the severity (S), and the probability of no detection for failure mode (D) were assigned to each failure mode by 8 professionals on a scale from 1 to 10. An overall risk priority number (RPN) for each failure mode was then calculated from the averaged O, S, and D scores. The coefficient of variation for each O, S, or D score was also calculated. The failure modes identified were prioritized in terms of both the RPN scores and the severity scores.

**Results:**

The established process tree for Gamma Knife radiosurgery consists of 10 subprocesses and 53 steps, including a subprocess for frame placement and 11 steps that are directly related to the frame‐based nature of the Gamma Knife radiosurgery. Out of the 86 failure modes identified, 40 Gamma Knife specific failure modes were caused by the potential for inappropriate use of the radiosurgery head frame, the imaging fiducial boxes, the Gamma Knife helmets and plugs, the skull definition tools as well as other features of the GammaPlan treatment planning system. The other 46 failure modes are associated with the registration, imaging, image transfer, contouring processes that are common for all external beam radiation therapy techniques. The failure modes with the highest hazard scores are related to imperfect frame adaptor attachment, bad fiducial box assembly, unsecured plugs/inserts, overlooked target areas, and undetected machine mechanical failure during the morning QA process.

**Conclusions:**

The implementation of the FMEA approach for Gamma Knife radiosurgery enabled deeper understanding of the overall process among all professionals involved in the care of the patient and helped identify potential weaknesses in the overall process. The results of the present study give us a basis for the development of a risk based quality management program for Gamma Knife radiosurgery.

## 1 INTRODUCTION

Gamma Knife radiosurgery is a high precision radiation therapy technique that can be used as an alternative or a complementary to open surgery for treating various brain disorders.^1^, ^2^, ^3^, ^4^, ^5^ Since the first clinical use of the Gamma Knife prototype at the Mottala workshop in Sweden, almost one million patients have been treated worldwide with different models of the Gamma Knife machines. Following Lars Leksell's original idea of stereotactic radiosurgery (SRS), linac and proton beam based SRS procedures for intracranial targets as well as stereotactic body radiotherapy (SBRT) procedures for body tumors were also developed.

A Gamma Knife radiosurgery procedure requires multidisciplinary efforts from a neurosurgery team, a radiation oncology team and other hospital based professionals. Ideally, the procedure should be performed in a comfortable environment for patient & staff with a high local control of the primary disease site and minimized radiation adverse effects to the normal tissue and the critical structures of consideration. To achieve this goal, continuous development of a system of reliable medical imaging modalities, accurate radiation therapy machines and effective treatment protocols is essential. Of equal importance is the implementation of a quality management program that ensures a safe and flawless execution of the treatment at the time of the procedures. Failure modes and effects analysis (FMEA)^6^ is a method that can be used as an important component of a comprehensive quality management program.

The FMEA is a reliability study tool for the analysis of the postulated component failures in a system and the resultant effects on the system operations.^7^ It was initially developed by the US military^8^ and has been extensively used in a variety of industries and health care services.^9^, ^10^, ^11^ The importance of a risk based quality management program for radiation therapy has come to the attention of the medical physics community in recent years owing to two facts. First, with the technology advances in equipment manufacturing and the development of various quality assurance protocols, conventional device specific physics QA measurements can be done with much higher precision than before. Second, many reported radiation therapy incidences resulted from incorrect or inappropriate use of radiation treatment devices due to miscommunication or misunderstanding rather than device failures. As such, the development of quality management programs that focuses on the design and execution of various radiotherapy processes has become a subject of significant interest in recent years.^12^, ^13^, ^14^, ^15^, ^16^, ^17^, ^18^, ^19^, ^20^ Following the recommendations from the AAPM task group report No. 100 (TG 100), the implementation of risk‐based quality management for radiation therapy facilities may become a standard practice and a regulatory requirement in the future.

As the first step toward the development of a risk‐based quality management program for Gamma Knife radiosurgery, we present in this work a FMEA study on the Gamma Knife radiosurgery process as performed at our institution following the methodologies described in the AAPM TG 100 report.

## 2 MATERIALS AND METHOD

### 2.A.1 Gamma Knife radiosurgery at our institution

More than 600 patients receive single fraction Gamma Knife radiosurgery treatments on a Leksell Gamma Knife Perfexion and a 4C at our institution annually. All the patients reported to the treatment suite at 5.30 a.m and were evaluated in one of the five exam rooms upon arrival. Each patient was cared for by a dedicated nurse during the entire treatment process. After the stereotactic coordinate frames were placed by the neurosurgical team shortly after 6.30 am, patients were transported to the radiology department for imaging. Neurosurgical team members supervised the MR, CT, or Angiographic imaging appropriate for the pathology undergoing radiosurgery. Morning QA of the treatment machines and patient chart creation was performed by physicists starting at 6.30 a.m. The radiosurgical planning using GammaPlan workstation took place as soon as the first set of images was available, usually started by the neurosurgical team and joined by the radiation oncology team. Final approval of the plan and plan export were done by physicists. The written directive was signed by an authorized surgeon, a radiation oncologist, and a medical physicist. The physicists also performed a secondary dose calculation check. The patients were then put on the treatment tables and docked into treatment positions. A patient identification check was performed in the presence of a physician, a physicist, and a nurse before the beam‐on. The first treatment on each machines usually started around 8.00 a.m. During the treatment times, a radiation oncologist, a physicist, and a nurse were present in the close vicinity of each treatment console. Frame removal was performed by the surgical and nursing team immediately after the treatment was finished. Patients were usually discharged within an hour from the time of frame removal.

### 2.A.2 Process tree and failure modes

The FMEA on the Gamma Knife radiosurgery was conducted following the methodology of the FMEA on IMRT as described in the AAPM TG 100 report. A team consisting of medical physicists, radiation oncologists, neurosurgeons, radiation safety officers, nurses, and schedulers at our institution and an external physicist expert on Gamma Knife was formed for the FMEA study. A preliminary process tree was prepared by the physics group first. Discussions about the details of each component of the treatment process between the physicists and other six professional groups were followed. The process tree was then revised and presented to all team members for further discussions and revision until a final version was agreed on.

The failure modes table was generated following the same path. A template with an initial version of the failure mode table was prepared by the physics group using the Microsoft Excel and distributed to other professional groups for revision and addition. Some potential failures could be detected and prevented by the treatment delivery system and were not included in the failure modes table. Examples of these failure modes include wrong collimator on the 4C, wrong Gamma angle on the Perfexion etc.

### 2.A.3 Scoring and risk prioritizing

The scoring process involved the medical physicists, two radiation oncologists, and three neurosurgeons who have been routinely involved in the Gamma Knife treatments. Three scores for the probability of occurrence (O), the severity (S), and the probability of no detection for failure mode (D) were assigned to each failure mode by each professional on a scale from 1 to 10. The methodology of the FMEA, the content of the Gamma Knife failure mode table and the scoring guidelines were discussed in detail before the scoring process. The guidelines used for the O, S, D scores were exactly the same as described in the TG 100 report. Averaged O, S, D scores and an overall risk priority number (RPN) were then calculated for each failure mode. To analyze the variation of the O, S, D scores from different scorers, coefficient of variation (defined as the ratio of standard deviation and mean) for the eight sets of data points was also calculated for each score.

The identified failure modes were analyzed in term of the RPN scores and the severity scores for risk prioritizing. The five failure modes with the highest RPN scores and the five failure modes with the highest severity scores were sorted out as targets for improvement.

## 3 RESULTS

**Figure 1:**
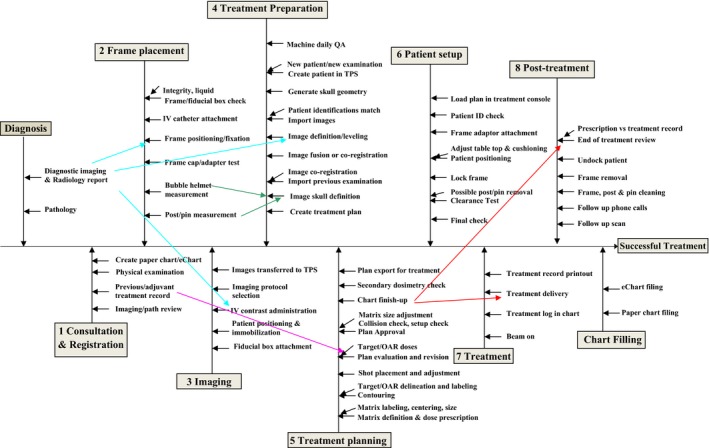
Process tree for Gamma Knife Radiosurgery as performed at our institution. The words after the angled arrows indicate the key components of the corresponding steps as described after the straight arrows.

Figure 1 shows the process tree for Gamma Knife radiosurgery as performed at our institution. A total of 10 subprocesses and 53 steps were identified for a Gamma Knife procedure starting from the diagnosis to the post‐treatment follow‐ups and chart filing. One subprocess (frame placement) and 11 steps are directly related to the frame‐based nature of Gamma Knife radiosurgery.

The neurosurgery team is essentially involved in all subprocesses of the process tree at our institution. The presence of the radiation oncology team during the frame placement and the imaging processes is not required. The role of the medical physicists starts from the treatment preparation subprocess.

Depending on the diagnosis and the location of the disease site, the course of a Gamma Knife treatment delivery process varies from patient to patient. On the Perfexion, Gamma angle change may be needed for some patients. On the 4C, helmet change, plug pattern, both the APS and the trunnion modes may be used for some patients. These details of the treatment delivery process were not documented in the process tree but were considered for the failure modes analysis.

Listed in Table 1 are the identified failure modes for Gamma Knife radiosurgery with the potential causes of the failure, the potential effects on patient/staff, examples of the failure modes and the steps in the process tree that the failure modes are related to. Also included in the table are the averaged O, S, D, and RPN scores for all failure modes. Out of the 86 failure modes identified, 40 failure modes are GK specific, caused by the potential for inappropriate use of the radiosurgery head frame, the imaging fiducial boxes, the GK helmets and plugs, and the GammaPlan treatment planning system. The other 46 failure modes are associated with the imaging, image transfer, image definition, contouring processes that are common for all external beam radiation therapy techniques.

**Table 1:** Failure modes table for Gamma Knife radiosurgery

No.	Step	Potential failure modes	Potential causes of failure	Potential effects of failure	O	S	D	RPN	Notes and examples of causes and failures
1	Create paper chart/eChart	Incorrect patient ID data	Errors in manual entry, most likely causes: 1. Omission in entry 2. Human transcription error 3. Miscommunication	Very wrong dose	2.00	8.00	1.50	24.00	Patient name is typed into the hospital database incorrectly. Information is requested from another department for a different person who actually exists. Information for the wrong patient is sent back. Suboptimal dose prescribed.
2	Previous/adjuvant treatment record	Incorrect or incomplete previous treatment history	1. Personnel omission 2. Miscommunication	Very wrong dose	2.00	6.13	2.50	30.63	Historical information about patient treatment inside/outside the dept. is inaccurate or incomplete. The patient & family don't remember treatment details.
3	Physical examination	Incorrect or incomplete test results	1. Lack of standardized procedures 2. Miscommunication 3. Personnel omission	Very wrong dose	2.50	3.25	1.75	14.22	Necessary physical or sensory tests are not done prior to the treatment, i.e. the most recent hearing test result is not available for an acoustic patient. Other test results may include vision, taste, motor function etc.
4	Physical examination	Incorrect or incomplete patient implant info	1. Lack of standardized procedures 2. Miscommunication	Patient injury	2.13	5.38	2.13	24.27	Patient has pacemaker, dental implant etc. but the information about the implant is not available or inaccurate at the time of the treatment. MR images were acquired instead of CT. Patient injured.
5	Frame/fiducial box check	Cannot start	1. Miscommunication 2. Personnel omission	Inconvenience	3.00	3.38	2.25	22.78	Patient does not show up because of a severe weather condition or a miscommunication between patient and staff. Patient shows up but finds out he/she is not on the schedule.
6	Frame/fiducial box check	Use of distorted frame	1. Lack of standardized procedures 2. Personnel omission	Inaccurate volume Inaccurate dose distribution delayed treatment	1.63	7.00	4.13	46.92	A distorted frame is used. The accuracy of the image definition/dose calculation is compromised. If the distortion is too much, patient can not be docked and the treatment can not proceed.
7	Frame/fiducial box check	Bad fiducial box assembly	1. Lack of standardized procedures 2. Personnel omission 3. Inadequate training/orientation	Inaccurate volume Inaccurate dose distribution Imaging may need to be repeated	3.38	4.88	3.75	61.70	A fiducial box is not assembled correctly, i.e. screws are loose, frontal/back or left/right pieces are switched etc.
8	Frame/fiducial box check	Too much air bubble	1. Lack of standardized procedures 2. Personnel omission	Inaccurate volume Inaccurate dose distribution	3.25	4.00	2.88	37.38	The length of the air bubbles in a MR imaging box should be minimized. Air bubbles in the fiducial box may cause certain image slices to be excluded from the image definition process.
9	Frame positioning/fixation	Inappropriate local anesthesia injection	Personnel inadequately trained	Patient uncomfortable	3.38	4.38	3.13	46.14	Local anesthesia not given to where pins will be inserted into skull bone.
10	Frame positioning/fixation	Frame not fixed firmly	1. Personnel omission 2. Personnel inadequately trained	Very wrong dose distribution Inconvenience	1.50	5.00	2.13	15.94	A frame is not attached to the patient skull firmly and becomes loose during the course of a treatment. The procedure has to start over if someone finds out about the loose frame before the treatment. Otherwise, treatment with a loose frame could mean a wrong dose distribution.
11	Frame positioning/fixation	Unsterilized pin/post	1. Lack of standardized procedures 2. Personnel omission	Patient infection	1.50	3.13	1.63	7.62	The use of unsterilized pin/post may cause infection to patient and/or staff.
12	Frame positioning/fixation	Inappropriate frame placement	1. Lack of training/experience 2. Miscommunication	Inconvenience (staff and patient)	2.25	6.50	1.88	27.42	The stereotactic frame is not shifted correctly when treating a peripheral lesion. A collision with the helmets (on the 4C) or the source cap (on the Perfexion) is reported from the planning system. Frame and imaging need to be repeated.
13	Frame cap/adapter test	Distorted frame adapter	1. Lack of standardized procedures 2. Personnel omission	Inconvenience (staff and patient)	1.75	4.88	1.75	14.93	The frame adapter for Perfexion is bad but not found out before a treatment. Treatment can not proceed.
14	Bubble helmet measurement	Inaccurate bubble measurement	1. Inadequate training of personnel 2. Human error	Inaccurate dose distribution	2.63	5.00	2.63	34.45	Bubble measurement is not accurate enough and is not noticed at the final check stage. Dose delivery is based on an inaccurate patient skull geometry.
15	Post/pin measurement	Incorrect post/pin measurement	1. Inadequate training of personnel 2. Human error	Inconvenience Patient injury	1.88	5.00	1.75	16.41	Post or pin measurement is not correct. A collision is not detected by the planning system. A collision with a post or pin could stop the treatment or injure the patient on treatment.
16	Fiducial box attachment	Fiducial box not attached properly	1. Inadequate training of personnel 2. Personnel omission	Inaccurate volume	2.50	3.88	2.25	21.80	The imaging fiducial box should be completely engaged with the frame and locked securely. Failure to do so may cause distortion in acquired images.
17	Patient positioning/immobilization	Unsecured imaging adaptor	1. Personnel inadequately trained 2. Inadequate materials/tools	Inaccurate volume Inaccurate dose distribution	1.63	8.63	2.75	38.54	Artifacts in images. Re‐imaging may be needed sometimes.
18	IV contrast administration	Contrast not used when needed	1. Inadequate communication from MD to sim staff 2. General procedures not clearly documented	Target volume not detected	2.13	4.38	4.25	39.51	Imaging contrast is not used appropriately. May cause inaccurate definition of the treatment volume.
19	Imaging protocol selection	Not enough imaging series	1. General procedures (e.g. all patients of a particular type should have certain series) not clearly documented 2. Miscommunication	Wrong target and/or OAR volume	1.75	6.38	2.38	26.50	Whole head study is not acquired, makes it difficult to check patient skull definition. Volumetric study is not acquired when needed, cause difficulty in target and/or critical organ delineation. Angio study is not acquired when needed.
20	Imaging protocol selection	Wrong scan protocol used (e.g. wrong slice thickness)	1. General procedures not documented 2. Miscommunication	Inaccurate volume inaccurate dose	2.38	5.38	2.63	33.51	Compromised CTV/OAR delineation and inaccurate dose delivery.
21	Imaging protocol selection	Patient move too much during scan	1. Poor communication to the patient 2. Imaging process too long	Inaccurate dose distribution	3.00	3.25	2.25	21.94	Patient may need to be rescanned.
22	Images transferred to TPS	Acquired image set accidentally deleted	1. Inattention to details 2. Inadequate training 3. Inadequate backup procedures	Inconvenience (staff and patient)	2.50	3.63	1.75	15.86	Imaging needs to be repeated. Inefficient imaging process.
23	Images transferred to TPS	Incorrect image data set associated with patient	1. Pull up wrong patient's record 2. Inadequate training	Very wrong volume Very wrong dose distribution	1.88	4.88	3.00	27.42	Patient treated on the basis of another patient's volumes.
24	Images transferred to TPS	Dicom communicating failure	1. Inadequate commissioning and acceptance testing 2. Limitations of treatment planning systems or scanners	Very wrong dose distribution Very wrong volume	1.38	7.63	2.25	23.59	A dicom communication failure causes corrupted files imported to the treatment planning system. Patient treated based on wrong information.
25	Images transferred to TPS	Can not transfer image via network	Defective materials (software or hardware)	Inconvenience Delayed treatment	1.88	6.00	1.50	16.88	Network problem not identified and fixed before a treatment. Images can not be transferred from a scanner to a treatment planning system. CD ROM may need to be used in some cases.
26	Machine daily QA	Staff not available	Miscommunication	Inconvenience Patient uncomfort Delayed treatment	3.00	1.63	2.63	12.80	After imaging, it has become clear that no or not enough coverage from the radiation oncology team is available. A patient treatment has to be cancelled after frame placement. A patient has to wait excessively long for a treatment.
27	Machine daily QA	Undetected console computer malfunctioning	1. Lack of standardized procedures 2. Personnel omission	Min: Inconvenience and delayed treatment Max: Very wrong dose	1.88	4.13	2.38	18.37	The treatment console computer is not working properly (timer, clock, system configuration etc.) but not identified during the morning QA process. Wrong clock or system configuration would prevent the system to start a treatment. Wrong timer would cause a wrong dose delivery.
28	Machine daily QA	Undetected mechanical failure	1. Lack of standardized procedures. 2. Human error	Inconvenience Delayed or partially completed treatment	2.50	5.38	3.63	48.71	There is a mechanical failure in the treatment unit (APS problem, helmet micro switch problem, helmet hoist malfunctioning, sector switch malfunctioning, couch problem etc.) but not identified during the morning QA process. Treatment can not start or may have to be stopped in the middle.
29	Create patient in TPS	Wrong category (new patient/new examination)	1. Personnel omission 2. Inadequate training	Min: Confusion and inconvenience Max: Very wrong dose	3.25	3.88	3.00	37.78	A patient underwent previous Gamma knife treatment is created as a new patient. The previous treatments could be ignored during the planning process.
30	Generate skull geometry	Wrong numbers (24 point bubble measurement)	Human error	Inaccurate dose distribution	2.13	4.00	2.75	23.38	Bubble measurement was done correctly but wrong numbers were put into the planning system. This is not noticed during the final check. Dose calculation is based on inaccurate patient skull geometry.
31	Generate skull geometry	Wrong numbers (Pin or post measurement)	Human error	Inconvenience Patient injury	3.25	4.38	2.13	30.21	Post or pin measurement is not put in correctly. A collision is not detected by the planning system. A collision with a post or pin could stop the treatment or injure the patient on treatment.
32	Import images	Images of another patient imported	Personnel omission	Very wrong dose	1.63	5.88	3.25	31.03	Images of a wrong patient were imported. The images of the two patients are similar, so the error was not discovered during the planning process. Treatment is based on wrong image sets.
33	Import images	Images of another patient imported	Personnel omission	Inconvenience	1.88	1.88	1.50	5.27	Images of a wrong patient were imported. The error was identified during the planning process. Treatment planning has to start over.
34	Import images	Incomplete image sets or image series	Personnel omission	Inconvenience	2.38	4.63	4.13	45.31	Miss an image set, i.e., forget to import T2 volumetric study. Start to import images before the image transfer is complete. Only part of the image set is imported. This can usually be found out during the planning process.
35	Image definition/levelling	Large definition error not noticed	1. Inadequate training 2. Personnel omission	Inaccurate volume Inaccurate dose distribution	3.38	4.00	3.25	43.88	The definition error for an image series is too big, either because of the use of bad equipment/protocol or excessive patient movement during the imaging process. Imaging needs to be repeated for optimal result if the large definition error is noticed.
36	Image definition/levelling	Mismatch between the bubble/image not noticed	1. Inadequate training 2. Personnel omission	Inaccurate dose distribution	3.13	3.75	2.00	23.44	The skull shape from the bubble measurement and the images don't match very well. Usually bubble measurement needs to be repeated.
37	Image definition/levelling	Wrong patient orientation information	1. Inadequate equipment commissioning 3. User Error	Wrong volume Suboptimal plan	1.75	4.63	2.88	23.27	This happens most often with the definition of Angio images. The frontal/back, left/right marks were not put in correctly.
38	Image definition/levelling	Inadequate leveling	Inadequate training	Wrong volumes	2.63	6.50	3.50	59.72	Images are not levelled appropriately. The boundary of a treatment area is blurred. Certain areas are overlooked because of poor image contrast.
39	Image fusion/registration	Can not get needed image sets	1. Inadequate equipment commissioning 2. Inadequate communication	Suboptimal plan	3.25	2.63	2.13	18.13	A pre‐op/post‐op image set is needed to evaluate the progression of a previous treated (radiosurgery or surgery) disease site but there are problems transferring the images from certain image server.
40	Import previous examinations	Wrong number of blue circles	1. Inadequate training 2. Personnel omission	Very wrong volumes Very wrong dose delivery	2.63	4.75	3.25	40.52	Forget to import one of the previous examinations. Forget to turn on some of the blue circles. Cause a previously treated area with good control to be considered as a new area and treated again.
41	Image skull definition	Skull not clean	1. Inadequate training 2. Personnel omission	Wrong dose distribution	2.88	3.88	2.50	27.85	The skull shape from an image skull definition is not manually edited properly. There are larger errors in certain areas of the skull contour.
42	Create treatment plan	Incorrect machine configuration	1. Inadequate commissioning 2. Personnel omission	Very wrong dose Very wrong dose distribution	1.63	3.13	3.50	17.77	Problems with the configuration of the treatment unit in the planning system. A wrong Gamma Knife unit is chosen (i.e., 4C vs Perfexion).
43	Create treatment plan	Incorrect algorithm configuration	1. Inadequate commissioning 2. Miscommunication 3. Personnel omission	Very wrong dose Very wrong dose distribution	2.75	3.00	2.00	16.50	Problem with the configuration of a dose calculation algorithm (wrong output factor, wrong CT density curve etc.).
44	Matrix definition/dose prescription	Wrong location of the targets	1. Inadequate training 2. Poor communication 3. Personnel omission	Very wrong volume Very wrong dose	2.50	3.75	2.38	22.27	Identification of the target area is not correct. Blood vessels, edemas are considered as tumors. Functional target left/right messed up, wrong location etc.
45	Matrix definition/dose prescription	Overlooked targets/target area	1. Inadequate training 2. Poor communication 3. Personnel omission	Ineffective treatment	1.75	4.50	2.63	20.67	Tumors or portions of tumors are not treated because of personnel omission or poor image quality.
46	Matrix definition/dose prescription	Inadequate prescription dose	1. Lack of standardized procedures. 2. Inadequate training 3. Poor communication	Very wrong dose	3.00	4.63	2.50	34.69	An inadequate prescription dose is used for a particular type of disease, depending on the experience of the planners, and/or the information gathered about the patient before the treatment.
47	Matrix definition/dose prescription	Inadequate matrix size/position	1. Lack of standardized procedures. 2. Inadequate training 3. Personnel omission	Inaccurate dose calculation	3.25	3.88	2.38	29.91	A dose calculation matrix is too sparse or not centered well. Dose calculation for this target is not accurate enough.
48	Shot placement & adjustment	Error in dose calc	Software error	Wrong dose distribution	1.38	6.50	1.63	14.52	Software cannot calculate dose correctly because of a computer problem. This can be checked by a secondary dose calculation program.
49	Contouring	Wrong organ, wrong site	1. Inadequate training 2. Personnel omission	Very wrong volumes	2.75	3.88	1.88	19.98	Wrong target volume or critical organ contours lead directly to very wrong dose distributions and volumes.
50	Contouring	Poorly drawn contours (spikes, sloppy, etc.)	Inattention	Suboptimal plan (worst case wrong dose distribution)	2.38	4.38	2.13	22.08	Drawings generally correct but have inappropriate spikes, sharp corners, etc. Plan evaluation is based on the imperfect contours. Wrong dose distribution in worst case.
51	Contouring/plan evaluation	Needed contour not drawn	1. Inadequate training 2. Inattention, lack of time 3. Failure to review own work	Suboptimal plan (worst case wrong dose distribution) Inconvenience	4.88	2.63	2.75	35.19	A critical structure is not drawn when needed. The structure is not included in plan evaluation. The plan is not optimal. Make it difficult to make clinical decisions for future treatments. Examples include skin dose is not evaluated, optical structure doses are not documented.
52	Plan evaluation	Inadequate evaluation	1. Not enough time/effort spent 2. Inadequate training 3. Poor evaluation strategy	Wrong dose Wrong dose distribution	1.63	6.88	2.38	26.53	One needs to look at DVH and dose distribution on a slice by slice basis. But because of poor training or not spending enough time to evaluate the DVH or isodose distribution, the result can be an inadequate evaluation of the plan.
53	Plan approval	Wrong patient	Inattention	Very wrong dose Very wrong dose distribution	1.75	6.50	4.13	46.92	Exported a treatment plan for a wrong patient. This happens rarely and can be found out in most of the cases.
54	Plan approval	Wrong plan approved	1. Miscommunication 2. Inattention 3. Inadequate procedure	Very wrong dose Very wrong dose distribution	2.25	5.88	3.63	47.92	Exported one of the trial plans rather than the final plan.
55	Plan approval	Matrix size not fine‐tuned	1. Inadequate procedure 2. Procedure not followed 3. Personnel omission	Inaccurate dose distribution	1.75	4.63	1.88	15.18	The sizes of the matrices are not adjusted appropriately before the plan is approved, resulting in inaccurate dose calculations.
56	Plan approval	Collision check not performed	1. Inattention 2. Lack of procedure 3. Procedure not followed	Inconvenience Patient injury	1.88	4.88	1.75	16.00	There is a collision between the helmet/cap and the patient skull, frame post/pin. Treatment may be interrupted and replan maybe needed. A collision with a pin or skull could injury the patient as well.
57	Plan approval	Setup not checked	1. Inattention 2. Lack of procedure 3. Procedure not followed	Inconvenience Suboptimal plan	2.63	5.00	2.50	32.81	Setup/shot summary is not checked. The exported plan contains an unwanted Gamma angle or helmet change, a shot with very short treatment time etc.
58	Chart finish‐up	Not signed appropriately	1. Inattention 2. Miscommunication	Inconvenience	3.25	2.25	1.75	12.80	The hard copy of the treatment plan is not signed appropriately. It could also mean that the final plan has not been checked and agreed by all planners in some cases.
59	Chart finish‐up	Unnoticed plugged pattern	1. Miscommunication 2. Lack of standard procedures	Very wrong dose Very wrong dose distribution Very wrong volume	1.88	5.13	2.13	20.42	A plug pattern is used on 4C but the hard copy does not show it, either because of an incorrect printout selection or a breakdown of the printer. Treatment proceeded without the plug pattern.
60	Secondary dosimetry check	Undetected planning computer failure	1. Lack of procedure 2. Inadequate procedure 3. Procedure not followed	Very wrong dose Very wrong dose distribution Very wrong volume	1.50	7.38	3.13	34.57	A failure in the treatment planning system gives an erroneous delivery time but this not caught by the second check. Or the secondary check is simply not done.
61	Plan export for treatment	Wrong plan exported	Personnel omission	Very wrong dose; Very wrong dose distribution Very wrong volume	1.13	9.13	1.75	17.96	This is highly unlikely for the recent versions of the planning system because only an approved plan can be exported. There are not many reasons to have several outstanding approved plans at a time in the Gamma Knife planning system.
62	Load plan in treatment console	Communication failure	1. Cable pulled out accidentally 2. Bad wire 3. Network problem	Inconvenience	2.13	1.88	2.63	10.46	The exported plan can not get to the treatment console computer. Treatment can not start. The communication between the planning computers and the treatment console computer is usually not checked during the morning QA.
63	Load plan in treatment console	Wrong plan loaded	Personnel omission	Very wrong everything	1.13	7.00	3.50	27.56	Load a plan for a different patient. This is also highly unlikely because there are not many reasons to keep more than one plan in the treatment queue.
64	Patient ID check	Incorrect patient in the room	1. Lack of standard procedures 2. Poorly trained personnel	Very wrong everything	1.88	3.75	2.50	17.58	Inadequate check.
65	Frame adapter attachment	Frame adapter not attached properly	1. Inadequate training/orientation 2. Personnel omission	Inconvenience Inaccurate dose delivery	2.75	7.75	5.75	122.55	On the Perfexion, the frame adaptor should be attached properly. Failure to do so may result in an interruption in the treatment process or an imperfect dose delivery. Elekta has a field notice about this.
66	Patient positioning	Couch vertical position not ideal	1. Lack of standard procedures 2. Inattention to detail. 3. Poorly trained personnel	Patient uncomfortable	2.25	3.75	3.50	29.53	The couch vertical position should be optimized so that the patient neck does not get stretched much. This is important for long treatments. The couch vertical position may need to be adjusted before each run. A different run means a different Gamma angle or tumors in a different area are being treated.
67	Lock patient	Incorrect patient setup	Personnel omission	Very wrong dose distribution	1.88	4.13	2.00	15.47	Patient setup with wrong stereotactic coordinates in the trunnion mode. Patient setup at a wrong Gamma angle. On the Perfexion treatment can not start with a wrong Gamma angle.
68	Lock patient	Patient head position not firmly fixed	1. Bad equipment 2. Personnel omission	Wrong dose distribution	2.63	3.63	2.50	23.79	It is good practice to check if the patient head is fixed properly before a treatment. Equipment failure, untighten or loose screws may cause unsecured patient head position. This is more important for the trunnion mode on the 4C.
69	Clearance test	Undetected collision	1. Personnel omission 2. Procedure not followed	Interrupted treatment Suboptimal plan Inconvenience Patient injury	1.50	6.50	1.75	17.06	A necessary clearance test is not performed or not performed properly. A treatment is interrupted because of a collision. Replan is needed. Patient may get injured.
70	Final check	Stuff on the couch	1. Personnel omission 2. Lack of standard procedure 3. Procedure not followed	Interrupted treatment	2.50	5.38	2.88	38.63	Pillows, blankets, screw drivers etc. are left on the couch. Treatment interrupted.
71	Final check	Tubes/cables not long enough	1. Personnel omission 2. Lack of standard procedure	Interrupted treatment Patient uncomfortable	2.88	2.75	3.50	27.67	A tube/wire (oxygen, pulse monitor, anesthesia equipment etc.) is attached to a patient. The tube/wire gets pulled when the couch moves into the treatment position. Treatment interrupted.
72	Final check	Wrong plug pattern	1. Personnel omission 2. Lack of double check	Wrong dose distribution	1.75	9.00	2.88	45.28	The plug pattern is not put in correctly on 4C.
73	Final check	Inserts/Plugs not secured	1. Personnel omission 2. Lack of double check	Interrupted treatment	3.25	5.13	3.63	60.38	An insert or plug is not firmly attached to a helmet. The insert/plug drops during a treatment. Cause the treatment to stop.
74	Final check	Bolus not used when needed.	1. Inadequate training 2. Inadequate procedure 3. Procedure not followed	Wrong or very wrong dose distribution	3.00	3.88	2.50	29.06	Bolus should be used for the treatment of superficial lesions. The surface portion of the lesion may be underdose if a bolus is not applied.
75	Beam on	Can not start treatment	1. Lack of training 2. Personnel omission	Delayed treatment	2.25	2.88	2.38	15.36	All the interlocks (couch release handle, side pieces, emergency buttons) has to be cleared before the beam‐on button lightens up. A patient is on the table but there is an un‐cleared interlock.
76	Beam on	Patient arms collide with machine	1. Lack of communication with patient 2. Inattention	Interrupted treatment Patient uncomfortable or injury	1.50	6.25	3.38	31.64	This happens most often when a restless or confused patient is being moved in or out of the treatment unit.
77	Treatment log book	Forget to record start/end time	Inattention	No effect on patient Inconvenience	2.50	2.25	2.25	12.66	Incomplete documentation.
78	Treatment monitoring	Treated shots not recorded correctly	1. Lack of standard procedure 2. Procedure not followed 3. Personnel omission	Very wrong dose Very wrong dose distribution	2.00	5.50	2.50	27.50	Delivered shots are not followed closely and recorded correctly on the treatment copy. Cause confusion in the shots that have been delivered. This happens more likely on 4C with trunnion shots.
79	Treatment monitoring	Patient hand reaches up	1. Lack of communication with patient 2. Inattention	Inaccurate dose delivery Interrupted treatment	3.00	2.63	3.38	26.58	Patient hands in the beam pathways. Cause underdose from some beam angles. Patient hand touch the source cap. Cause the treatment to be interrupted.
80	Treatment monitoring	Inappropriate sedation	Lack of experience/training	Patient comfort	1.88	6.75	1.88	23.73	A patient is overly sedated. Cause the treatment to be interrupted.
81	Treatment monitoring	Anesthesia problem	Lack of experience/training	Interrupted treatment	2.75	2.75	1.75	13.23	A patient is treated under general anesthesia. But an anesthesia problem cause a treatment to be interrupted.
82	Treatment monitoring	Poor choice of treatment sequence	Poor user choice	Ineffective treatment process	2.00	4.88	2.00	19.50	On 4C, when multiple helmets, multiple Gamma angles, high/low docking are involved, it is necessary to figure out a treatment sequence that minimizes the treatment time.
83	Treatment monitoring	Manmade treatment interruption	Inattention	Interrupted treatment	2.00	1.63	3.13	10.16	During the course of a treatment, someone accidently press the power button, door open, emergency, pause button, or spill liquids on the treatment console computer, cause the treatment to be interrupted.
84	Treatment monitoring	Unfinished treatment	Personnel omission	Very wrong dose	2.38	4.63	2.63	28.83	A treatment run is completely ignored.
85	Follow up phone calls	Unaddressed patient concerns	Inadequate procedure Personnel omission	Patient comfort	1.88	1.75	1.75	5.74	Patient has question to ask after Gamma Knife treatment, but is not answered appropriately.
86	Follow up scan	Follow up scan not at the right time	Miscommunication to patient	Patient care	2.50	1.88	1.63	7.62	May cause delay in further treatment.

The majority of the failure modes are not related to the configuration of the treatment device and are common for the Perfexion and the 4C. Table 2 lists the 10 failure modes that are treatment machine dependent. Failure modes No. 13 “distorted frame adaptor” and No. 65 “frame adaptor not attached properly” are related to the handling of the frame adaptor on the Perfexion. Failure modes No. 59 “unnoticed plugged pattern”, No. 72 “wrong plug pattern”, No. 73 “inserts/plugs not secured”, and No. 82 “poor choice of treatment sequence” are related to the use of plugs and helmets on the 4C. Failure modes No. 12 “inappropriate frame placement”, No. 67 “incorrect patient setup”, No. 68 “patient head position not firmly fixed”, and No. 78 “treated shots not recorded correctly” are shared by both machines but more commonly seen on the 4C, especially with the trunnion mode of the 4C.

**Table 2:** Failure modes that are treatment machine dependent

No.	Step	Potential failure modes	Potential causes of failure	Potential effects of failure	Machine	Notes and examples of causes and failures
13	Frame cap/adapter test	Distorted frame adapter	1. Lack of standardized procedures 2. Personnel omission	Inconvenience (staff and patient)	Perfexion	The frame adapter for Perfexion is bad but not found out before a treatment. Treatment can not proceed.
65	Frame adapter attachment	Frame adapter not attached properly	1. Inadequate training/orientation 2. Personnel omission	Inconvenience Inaccurate dose delivery	Perfexion	On the Perfexion, the frame adaptor should be attached properly. Failure to do so may result in an interruption in the treatment process or an imperfect dose delivery. Elekta has a field notice about this.
59	Chart finish‐up	Unnoticed plugged pattern	1. Miscommunication 2. Lack of standard procedures.	Very wrong dose Very wrong dose distribution Very wrong volume	4C	A plug pattern is used on 4C but the hard copy does not show it, either because of an incorrect printout selection or a breakdown of the printer. Treatment proceeded without the plug pattern.
72	Final check	Wrong plug pattern	1. Personnel omission 2. Lack of double check	Wrong dose distribution	4C	The plug pattern is not put in correctly on 4C.
73	Final check	Inserts/Plugs not secured	1. Personnel omission 2. Lack of double check	Interrupted treatment	4C	An insert or plug is not firmly attached to a helmet. The insert/plug drops during a treatment. Cause the treatment to stop.
82	Treatment monitoring	Poor choice of treatment sequence	Poor user choice	Ineffective treatment process	4C	On 4C, when multiple helmets, multiple Gamma angles, high/low docking are involved, it is necessary to figure out a treatment sequence that minimizes the treatment time.
12	Frame positioning/fixation	Inappropriate frame placement	1. Lack of training/experience 2. Miscommunication	Inconvenience (staff and patient)	Both	The stereotactic frame is not shifted correctly when treating a peripheral lesion. A collision with the helmets (on the 4C) or the source cap (on the Perfexion) is reported from the planning system. Frame and imaging need to be repeated.
67	Lock patient	Incorrect patient setup	Personnel omission	Very wrong dose distribution	Both	Patient setup with wrong stereotactic coordinates in the trunnion mode. Patient setup at a wrong Gamma angle. On the Perfexion treatment can not start with a wrong Gamma angle.
68	Lock patient	Patient head position not firmly fixed	1. Bad equipment 2. Personnel omission	Wrong dose distribution	Both	It is good practice to check if the patient head is fixed properly before a treatment. Equipment failure, untighten or loose screws may cause unsecured patient head position. This is more important for the trunnion mode on the 4C.
78	Treatment monitoring	Treated shots not recorded correctly	1. Lack of standard procedure 2. Procedure not followed 3. Personnel omission	Very wrong dose Very wrong dose distribution	Both	Delivered shots are not followed closely and recorded correctly on the treatment copy. Cause confusion in the shots that have been delivered. This happens more likely on 4C with trunnion shots.

**Figure 2:**
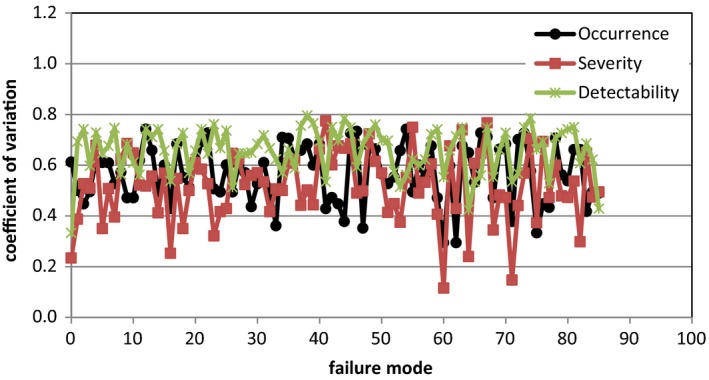
Coefficient of variation for the eight sets of occurrence, severity, and detectability scores.

Figure 2 shows the coefficient of variation for the 8 sets of the O, S, D scores for each failure mode. The averaged coefficients of variation for the O, S, D scores of the 86 failure modes are 0.57, 0.52, and 0.66 respectively. The largest coefficients of variation for the O, S, D scores are 0.74, 0.77, and 0.8 for failure modes No. 13 “distorted frame adaptor”, No. 68 “patient head position not firmly fixed”, and No. 39 “cannot get needed image sets” respectively. The smallest coefficients of variation for the O, S, D scores are 0.29, 0.12, and 0.33 for failure modes No. 61 “wrong plan exported”, No. 61 “wrong plan exported”, and No. 1 “incorrect patient ID data” respectively. The coefficients of variation for the severity scores are in general smaller than those for the occurrence and the detectability scores, indicating better agreement among the observers on the severity of the identified failure modes.

Table 3 lists the five failure modes with the highest RPN scores. Failure mode No. 65 “frame adaptor not attached properly” has the highest RPN of 122.55 and also the highest detectability score of 5.75. The manufacturer Elekta AB (Stockholm, Sweden) had issued a field notice about the potential problem with the inappropriate use of the frame adapter on the Perfexion and the high probability of this failure going undetected.^21^ Failure mode No. 73 “inserts/plugs not secured” is specifically related to the handling of the plug/helmets on the 4C. Failure modes No. 7 “bad fiducial box assembly” and No. 38 “inadequate leveling” are imaging related failures. Failure mode No. 28 “undetected mechanical failure” is a device specific physics QA issue.

**Table 3:** Failure modes with the highest RPN scores for Gamma Knife Radiosurgery

No.	Step	Potential failure modes	Potential causes of failure	Potential effects of failure	RPN	Notes and examples of causes and failures
65	Frame adapter attachment	Frame adapter not attached properly	1. Inadequate training/orientation 2. Personnel omission	Inconvenience Inaccurate dose delivery	122.55	On the Perfexion, the frame adaptor should be attached properly. Failure to do so may result in an interruption in the treatment process or an imperfect dose delivery. Elekta has a field notice about this.
7	Frame/fiducial box check	Bad fiducial box assembly	1. Lack of standardized procedures 2. Personnel omission 3. Inadequate training/orientation	Inaccurate volume Inaccurate dose distribution Imaging may need to be repeated	61.70	A fiducial box is not assembled correctly, i.e. screws are loosen, frontal/back or left/right pieces are switched etc.
73	Final check	Inserts/plugs not secured	1. Personnel omission 2. Lack of double check	Interrupted treatment	60.38	An insert or plug is not firmly attached to a helmet. The insert/plug drops during a treatment. Cause the treatment to stop.
38	Image definition/levelling	Inadequate leveling	Inadequate training	Wrong volumes	59.72	Images are not levelled appropriately. The boundary of a treatment area is blurred. Certain areas are overlooked because of poor image contrast.
28	Machine daily QA	Undetected mechanical failure	1. Lack of standardized procedures. 2. Human error	Inconvenience Delayed or partially completed treatment	48.71	There is a mechanical failure in the treatment unit (APS problem, helmet micro switch problem, helmet hoist malfunctioning, sector switch malfunctioning, couch problem etc.) but not identified during the morning QA process. Treatment can not start or may have to be stopped in the middle.

Table 4 gives the 5 failures with the highest severity scores. Failure mode No. 72 “wrong plug pattern” is related to the inappropriate use of the plug pattern on the 4C. Failure mode No. 65 “frame adaptor not attached properly” is found on both the riskiest and severest lists and is the primary target of our risk‐based quality assurance program.

**Table 4:** Failure modes with the highest severity scores for Gamma Knife Radiosurgery

No.	Step	Potential failure modes	Potential causes of failure	Potential effects of failure	S	Notes and examples of causes and failures
61	Plan export for treatment	Wrong plan exported	Personnel omission	Very wrong dose Very wrong dose distribution Very wrong volume	9.13	This is highly unlikely for the recent versions of the planning system because only an approved plan can be exported. There are not many reasons to have several outstanding approved plans at a time in the Gamma Knife planning system.
72	Final check	Wrong plug pattern	1. Personnel omission 2. Lack of double check	Wrong dose distribution	9.00	The plug pattern is not put in correctly.
17	Patient positioning/immobilization	Unsecured imaging adaptor	1. Personnel inadequately trained 2. Inadequate materials/tools	Inaccurate volume Inaccurate dose distribution	8.63	Artifacts in images. Re‐imaging may be needed sometimes.
1	Create paper chart/eChart	Incorrect patient ID data	Errors in manual entry, most likely causes: 1. Omission in entry 2. Human transcription error 3. Miscommunication	Very wrong dose	8.00	Patient name is typed into the hospital database incorrectly. Information is requested from another department for a different person who actually exists. Information for the wrong patient is sent back. Suboptimal dose prescribed.
65	Frame adapter attachment	Frame adapter not attached properly	1. Inadequate training/orientation 2. Personnel omission	Inconvenience Inaccurate dose delivery	7.75	On the Perfexion, the frame adaptor should be attached properly. Failure to do so may result in an interruption in the treatment process or an imperfect dose delivery. Elekta has a field notice about this.

## 4 DISCUSSION

A single fraction Gamma Knife procedure is designed to be a fast and efficient radiation delivery process that can be completed (frame on to frame off) within a few hours. Comparing to other radiosurgery techniques, the use of the stereotactic head frame for patient positioning also helps to eliminate many uncertainties at the imaging, the target delineation, and the treatment setup stages.^22^

Seventy six of the 86 failure modes found in this study are independent of the Gamma Knife treatment units and are common for the Perfexion and the 4C. Two failure modes (No. 13 and No. 65) were found specific for the Perfexion and 4 failure modes (No. 59, No. 72, No. 73, No. 82) were found specific for the 4C. Even though slightly fewer failure modes were identified for the Perfexion, the failure mode with the inappropriate use of the frame adaptor on the Perfexion clearly stands out in terms of the risk priority number.

The statistically large coefficients of variation for the 8 set of scores collected indicate substantial observer dependent differences in the understanding of the scoring guidelines and/or the interpretation of the failure modes. The differences in the perspectives of different professional group on certain failure modes may also contribute to the variations. The variations in the detectability scores are in general larger comparing to the other two groups, partly because detectability is a brand new concept that comes with the FMEA. Generally speaking, the statistic aspect of the scores could be improved by increasing the number of qualified scorers in the study group.

The top five failure modes with the highest RPN scores and the highest severity scores were used as the starting point for the development of a risk based quality management program. Failure mode No. 65 “frame adaptor not attached properly” was found on both the riskiest list and the severest list and was of top priority for quality improvement consideration. On the Perfexion Gamma Knife unit, the accuracy of the radiation dose delivery relies heavily on the appropriate use of the frame adaptor, which is the only component between the patient head frame and the robotic patient positioning system. Unlike the previous models of the Leksell Gamma Knife, patient positioning on the Perfexion is fully automated and the stereotactic coordinates for each shot cannot be visually verified by human eyes. Therefore, every effort should be made to ensure that all patients are attached to the treatment couch perfectly and secured in position during the course of their treatment because of the high detectability score of this failure mode. As per our QA policy, the proper functioning of the frame adaptor should be checked by a physicist during the morning QA process in conjunction with the focus precision diode test. The frame adapter should be applied to any patients by a clinical staff member according to the following rules: (a) always assure that the three metal latches are at a right angle with the frame adapter; (b) never force the plastic lever in place if it meets significant resistance when turned; (c) ensure that the plastic lever is completely flush with the frame adapter and that no angulations are present. As a result of this study, we implemented a policy that requires a physicist to visually check the gap between the frame and the frame adaptor, the position of the lock and the latches before each treatment. The physics staff is also required to apply some force to the frame adaptor to ensure that it is firmly attached to the treatment couch. We also communicated with the manufacturer about the result of this investigation and emphasized from the user point of view the importance to refine the locking/docking mechanism of the frame adaptor.

Our physics QA process was reviewed and revised to address failure modes No. 28 “undetected mechanical failure” and No. 7 “bad fiducial box assembly”. On the Perfexion, the docking of the frame adapter at the 70, 110 degree Gamma angles was added to the morning QA check in addition to the 90 degree gamma angle. On the 4C, the frequency of the helmet micro‐switch tests for all the four helmets was changed to weekly from monthly. A step was also added to the physics morning QA procedure to check the integrity of all the image boxes to be used, including the liquid in the MR fiducial box, the fiducials and the orientation markers on the CT/Angio boxes.

Failure modes No. 73 “inserts/plugs not secured” is related to the use of plugs on the Gamma Knife 4C for certain treatments. A plug pattern is usually generated for a specific helmet during the planning process and all the plugs are subsequently put in by a clinical staff member manually according to the treatment report printout. Previously our protocol was to have a second clinical staff member to visually verify the plug pattern and check the firmness of the plug positions using his/her hands. To reduce the occurrence of this failure mode, a second round of checks by a third staff member was added.

To reduce the potential damage from failure mode No. 38 with inadequate image leveling, a mandatory neuroimaging in‐service on the basics of the MR, CT, and the Angio imaging techniques was given to all clinical staff. A policy was also developed to require at least one T1 weighted image series and one T2 weighted image series for any MR based treatments, so that targets and critical structures can be drawn on different image sets and cross‐checked when necessary.

Several of the failure modes with the highest severities scores did not get high RPN scores because of their low scores in both the occurrence and the detectability. The reason was that many QA procedures had already been developed and implemented over the years to prevent these dangerous failure modes from happening. These procedures include patient ID double check, plug pattern check, and image adaptor position check etc.

It should be pointed out that the present study was conducted for the Gamma Knife radiosurgery procedures as performed at our institution. The results of the risk prioritizing process, the details of the failure mode table, and even the layout of the process tree might be different for other institutions, even though the bulk part of the FMEA should be the same for all Gamma Knife facilities.

## 5 CONCLUSIONS

We have performed a FMEA study on Gamma Knife radiosurgery based on our experience with the treatments of more than 600 patients annually. The implementation of the FMEA approach was first an important self‐learning process that enabled deeper understanding of the radiosurgery procedure among all professionals involved in the care of the patient. The identified weaknesses in the overall process were the primary target areas for the development of a risk based quality management program for Gamma Knife radiosurgery.

## CONFLICT OF INTEREST

The authors declare no conflict of interest.
